# Characteristics of Chinese patients with cough in primary care centre

**DOI:** 10.1186/1479-5876-9-149

**Published:** 2011-09-10

**Authors:** Qunying Hong, Chunxue Bai, Xiangdong Wang

**Affiliations:** 1 Department of Pulmonary Medicine, Fudan University Zhongshan Hospital, Shanghai, 200032, China; 2 Biomedical Research Center, Fudan University Zhongshan Hospital, Shanghai, 200032, China

## Abstract

**Background:**

Cough is one of the most common respiratory symptoms and is well characterized in specialized cough clinics with high success rates of diagnosis and treatment. However, there is a paucity of data regarding cough in primary care settings. The present study aimed at investigating clinical epidemiology of cough through a national study of two questionnaire surveys sent to primary care physicians in China.

**Methods:**

Approximately 18,000 subjects recruited were having daytime or night symptoms of cough and diagnoses of respiratory disease from February 2005 to April 2006 as Survey 1 and from June 2007 to December 2007 as Survey 2. Patients suffering from respiratory malignancy, hyperthyroidism, hypertension, heart disease, diabetes, severe hypohepatia or renal dysfunction, pregnancy, possible pregnancy or lactation, neutropenia were not eligible. Information regarding demography, history of allergies, symptomatic profile, treatment and curative effects for cough was elicited.

**Results:**

8216 questionnaires were collected in Survey 1 and 9711 in Survey 2. The mean values of ages were 25.7 and 22.3 years old, respectively. Symptoms included expectoration (74% and 76%), wheeze (59% and 74%), breathlessness (22% and 26%), chest pain (9% and 13%) and fever (15% and 18%). About 15% and 23% patients had hypersusceptibility, of whom 6% to 17% had a family history. More than 50% of the cases had histories of allergic rhinitis, asthma, conjunctivitis or atopic dermatitis. Asthma, COPD, and bronchitis were dominant etiologies of cough. Procaterol or the combination of antibiotics and steroids were used as the treatment.

**Conclusion:**

Causes and outcomes of cough differed with ages and time in this particular national study, while successful and precise diagnosis and management of cough in primary care settings need to be further improved in China.

## Introduction

Cough is one of the most common respiratory symptoms encountered by clinicians [1], with the reported prevalence varying between 5% and 40% [2-4]. Cough is also one of the critical factors affecting the quality of life in patients with respiratory diseases. Cough management has massive economic consequences. The first guideline on the management of cough with a significant positive impact was championed in 1998 [5], followed by the publication of other guidelines on chronic cough [6-9]. The Chinese Medical Association published "Diagnosis and treatment guide (draft) of cough" in 2005 [10], and then the American College of Chest Physicians published evidence-based guidelines for diagnosis and treatment of acute cough, subacute cough, and chronic cough in adults and chronic cough in children in 2006 [1,6,11].

Over-the-counter combination cough medication such as cough syrup including traditional Chinese medicine is usually the first choice in China. The majority of people with cough who consulted a health care professional received the diagnosis and was informed that over the counter cough medications could relieve the symptoms. However, there are still a number of those with unclear diagnosis or failure to respond to therapy. The present study aimed at investigating clinical epidemiology of cough through a national study of two questionnaire surveys sent to primary care physicians in China. Approximately 18,000 subjects with daytime or night symptoms of cough and with diagnoses of respiratory disease were recruited for questionnaires from February 2005 to April 2006 as Survey 1 and from June 2007 to December 2007 as Survey 2. It provided further information about the demographic and symptomatic profile in the population and curative effects for cough in Chinese patients with respiratory diseases. This is one of the national projects organized by the Chinese Medical Doctor Association in order to obtain a better understanding of coughs while hoping the results could lead to new ideas and plans to explore in regard to molecular mechanisms and therapies.

## Methods

### Patients

The present study identified clinical characteristics of patients with cough from a nationwide-based survey conducted by the Chinese Medical Doctor Association. Subjects recruited had daytime or nighttime symptoms of cough and diagnoses of respiratory diseases, from 85 primary care medical centers. The 85 clinics are primary care centers chosen from the north and south of China as representatives in China. One survey started from February 2005 to April 2006 was named Survey 1 and one from June 2007 to December 2007 was named Survey 2. The two surveys were performed at the same 85 clinics. There was no overlap of the two surveys. The reason for calculating using two surveys was to see the improvement of health care and medical care inbetween the two time periods. Patients were provided with written information prior to obtaining oral consent. The study is a questionnaire survey aimed at investigating the clinical epidemiology of the cough for patients when first visiting the primary care center. Information on patient health care data, e.g. numbers, home address, phone number and others, was protected.

### Evaluation Scores

Physicians were invited to complete and follow up the cough questionnaires and were then completed by the referred patients who had been in primary care settings during the first time. After then, they were continuously followed up for an additional 14 days, in order to audit social and demographic factors as well as qualitative measures of responses to treatment. The physicians filled out the questionnaires. Sections of open questions were asked, including demographic details, symptoms, history of the cough, hypersusceptibility patterns, and treatment outcomes. Hypersusceptibility was defined as a cough condition of abnormal susceptibility to stimuli that are entirely innocuous in the normal individual. Cough severity was assessed on a scale of 0 (no cough) to 3(severe cough) and recorded on a daily record. Effectiveness was evaluated as cured (symptom score decreased to 0), markedly improved (symptom score decreased ≥ 4), improved (Symptom score decreased ≥ 2), or no effect (unchanged or aggravated). Adverse events were elicited at each visit by enquiring about the side effects of therapy recorded from a symptom checklist.

### Statistics

Data were analyzed by the two-sided test and p value less than 0.05 was considered significant. Non-parametric statistics rank sum test was used for the analysis of recovery percentage. The quota target was used and described by statisticians, including the computation arithmetic mean value, the standard deviation, the median, the minimum value, the maximum value. The example number was calculated and the percentage of corresponding was classified with the identification target. Data was analyzed using the SAS 9.1.2 program by Health Statistics Laboratory of Chinese Center for Disease Control and Prevention.

## Results

Of 18000 questionnaires, 8216 were received by the end of April, 2006, and 9711 by the end of Dec 2007, respectively. There were 4655 males and 3561 females with a mean age of 25.7 (standard deviation 24, range 1-98 years) in Survey 1 with 8216 patients. Of them, 46% were less than 10 years old (Table 1 and Table 2). There were 5331 males and 4010 females with a mean age of 22.3 years (standard deviation 22 years) in Survey 2 with 9711 patients, among whom the age of 425 patients were not recorded.

**Table 1:** Characteristic of the subjects in Survey 1

Age	Male		Female		Total	
Years	Cases	Percentage	Cases	Percentage	Cases	Percentage
0-10	2102	45.2%	1710	48.0%	3812	46.4%
11-20	249	5.3%	196	5.5%	445	5.4%
21-30	279	6.0%	281	7.9%	560	6.8%
31-40	519	11.1%	397	11.1%	916	11.2%
41-50	492	10.6%	299	8.4%	791	9.6%
51-60	382	8.2%	239	6.7%	621	7.6%
61-70	321	6.9%	151	4.2%	472	5.7%
71-80	212	4.6%	107	3.0%	319	3.9%
81-90	99	2.1%	181	5.1%	280	3.4%
Total	4655	100%	3561	100%	8216	100%

**Table 2:** Characteristic of the subjects in Survey 2

Age	Male		Female		Total	
	Cases	Percentage	Cases	Percentage	Cases	Percentage
0-10	2439	45.5%	2252	55.8%	4691	50.5%
11-20	812	15.2%	539	13.4%	1351	14.5%
21-30	216	4.2%	175	4.2%	391	4.2%
31-40	426	8.1%	269	6.6%	695	7.5%
41-50	535	9.9%	249	6.2%	784	8.4%
51-60	361	6.7%	208	5.1%	569	6.1%
61-70	297	5.5%	154	3.8%	451	4.9%
71-80	201	3.8%	132	3.2%	333	3.6%
81-90	14	0.2%	7	0.1%	21	0.2%
Total	5301	100%	3985	100%	9286	100.0%

The cough was reported to be productive in 6256 of 8216 patients (76%) with complaint of wheeze 59%, breathlessness 22%, chest pain 9% and fever 18% in Survey 1, while cough (74%), wheeze (74%), breathlessness (26%), chest pain (13%) and fever (15%) were noticed in Survey 2. Of 8216 patients in Survey 1, 1919 (23%) had hypersusceptibility and 1359 (17%) had a family history of hypersusceptibility. Of 9177 patients in Survey 2, 1444 (15%) had hypersusceptibility and 621 (6%) had a family history of hypersusceptibility. More than 50% of cases had a history of allergic rhinitis, asthma, allergic conjunctivitis or atopic dermatitis (Table 3). Inclusion subjects were given a diagnosis, mainly including COPD and asthma. Table 4 described the details of diagnosis. Tubercular pleurisy, stomatitis, acute pharyngitis, hypertension of pulmonary artery or fewer were listed in the section of others.

**Table 3:** History of diseases in subjects with cough

Disease	Survey 1		Survey 2	
	Cases	Percentage	Cases	Percentage
Allergic rhinitis	1359	16.5%	1972	20.3%
Asthma	3202	39.0%	6236	64.2%
Allergic conjunctivitis	88	1.1%	227	2.3%
Atopic dermatitis	120	1.5%	104	1.1%

**Table 4:** Diagnosis of inclusion subjects

Diagnosis	Survey 1		Survey 2	
	Cases	Percentage	Cases	Percentage
Influenza	2	0.02%	1	0.01%
Rhinitis	28	0.34%	30	0.31%
Bronchial hyperreactivity	19	0.23%	0	0.00%
COPD & Asthma	90	1.10%	0	0.00%
Chronic bronchitis	124	1.51%	105	1.08%
Pneumonia	290	3.53%	87	0.90%
Allergic cough	334	4.07%	301	3.10%
Asthmatoid bronchitis	341	4.15%	292	3.01%
Common cold	394	4.80%	31	0.32%
Chronic cough	520	6.33%	249	2.56%
Acute bronchitis	932	11.34%	1000	10.30%
Asthma (except CVA)	1799	21.89%	4521	46.56%
COPD	1303	15.86%	1135	11.69%
Cough variant asthma(CVA)	1723	20.97%	1563	16.10%
others	317	3.86%	400	4.08%

**Total**	8216	100.00%	9711	100.00%

Patients were treated with Procaterol, of whom 1098 in Survey 1 and 1046 in Survey 2 also had a combination of drugs, including expectorant (Ambroxol, Bromhexine, Carbocisteine), methylxanthines (Aminophylline, Theophylline), antihistamines (Cetirizine, Ketotifen, Cyprohentadine, Loratadine, Chlorphenamine, Tranilast), leukotriene modifiers (Montelukast), beta2 agonists (Salbutamol), inhaled long-acting beta2 agonists, Sametrol/Fluticasone, Formoferol/Budesonide, anticholinergics (Ipratropine). Combined drugs also included cough syrup (e.g. graifenesin, methylephedrine, chlorphenamine syrup, dextromethorphan hydrobromide with chlorpheniramine maleate, or pseudoe phedrine hydiochloride solution), Asmeton, Chinese traditional medicine, inhaled steroids (e.g. budesonide, fluticasone, and beclomethasone dipropionate), systemic steroids (e.g. methylprednisolone, prednisone, and dexamethasone), anti-virus (Ribavirin), or antibiotics(e.g. macrocyclic lactone, quinolone, cephalosporin, benzylpenicillin, and others), as shown Table 5.

**Table 5:** Treatments of inclusion subjects

Treatment		Survey 1		Survey 2	
		
		Cases	Percentage	Cases	Percentage
Expectorant		194	2.36%	1	0.01%
Methylxanthines		126	1.53%	39	0.40%
Antihistamines		197	2.40%	48	0.49%
Leukotriene modifiers		104	1.27%	151	1.55%
SABAS		43	0.52%	184	1.89%
LABA		34	0.41%	53	0.55%
Anticholinergics		36	0.44%	2	0.02%
Combination Drugs		82	1.00%	1	0.01%
Chinese traditional medicine		169	2.06%	14	0.14%
Inhaled steroids		180	2.19%	225	2.32%
Systemic Corticosteroids		45	0.55%	22	0.23%
Anti-virus		6	0.07%	0	0.00%
	Macrocyclic lactone	88	1.07%	5	0.05%
	Quinolone	51	0.62%	0	0.00%
Antibiotics	Cephalosporin	267	3.25%	243	2.50%
	Benzylpenicillin	45	0.55%	31	0.32%
	others	12	0.15%	72	0.74%
Others drugs (unknown)		414	5.04%	19	0.20%

The daily cough diary showed a rapid and highly significant reduction in the cough score (Table 6 and Table 7). Of them, 83% in Survey 1 and 92% in Survey 2 had no symptom during the daytime after 14 days of treatment, while 82% in Survey 1 and 94% in Survey 2 had no symptom during the night. There was a significant difference between before and after the treatments (p < 0.05 or 0.01, respectively). No patient dropped out because of severe adverse events.

**Table 6:** Daytime cough symptom assessment

Days	Cases in survey 1				Cases in survey 2			
	
	No symptom	Mild cough	Moderate cough	Severe cough	No symptom	Mild cough	Moderate cough	Severe cough
0	34	807	3146	3926	12	182	2561	6463
1	35	701	2978	3617	7	206	2475	5764
2	87	910	3493	2903	69	557	3337	4568
3	218	1291	4075	1890	227	1016	4371	3156
4	366	1671	4495	846	145	1426	6034	673
5	521	2070	4326	413	309	2390	5394	173
6	805	2692	3682	183	633	3342	4229	65
7	1151	3482	2896	76	1326	3777	3473	42
8	1449	4035	1756	41	890	4810	1614	29
9	1895	4250	1077	24	1438	5065	647	26
10	2382	4199	605	13	2240	4401	251	20
11	3166	3075	304	5	3058	3617	102	19
12	4370	2655	123	7	4758	1859	48	19
13	5280	1799	62	4	5056	966	33	19
14	6083	1388	46	5	5540	704	33	19

**Table 7:** Night cough symptom assessment

Days	Cases in survey 1				Cases in survey 2			
	
	No symptom	Mild cough	Moderate cough	Severe cough	No symptom	Mild cough	Moderate cough	Severe cough
0	149	819	2939	3942	43	239	2471	6441
1	80	818	2632	3756	39	272	2424	5690
2	167	944	2967	3165	88	615	2830	4971
3	318	1190	3382	2349	236	1110	4274	3132
4	466	1512	3944	1310	171	1467	5455	1159
5	640	1800	4126	637	339	2198	5420	296
6	895	2223	3826	287	555	3124	4483	92
7	1276	2780	3272	133	1379	3777	3423	39
8	1513	3180	2373	68	978	4664	1747	23
9	1809	3655	1605	28	1395	4759	1091	19
10	2151	3944	936	14	2048	4518	392	15
11	2704	3809	495	4	3096	3591	132	15
12	3711	3077	214	1	4651	1983	48	17
13	4778	2121	87	1	5030	998	28	17
14	5903	1417	48	0	5716	531	24	18

## Discussion

Cough is an important defensive reflex of the respiratory tract to clean up and protect the upper airway, while also being the commonest symptom for which patients seek medical advice. Causes and outcomes of cough differ with age and its presentation and other related symptoms vary with time (e.g. seasonality) and habits (e.g. smoking, occupation, body weight, use of drug etc.). Children below 10 years may have more episodes of bronchiolitis and asthma, which is the reason why their prevalence is high among both surveys in the present, while the adults suffer more from pneumonia and other chronic conditions. One of the common causes is chronic upper airway cough syndrome, previously referred to as postnasal drip syndrome, secondary to rhinosinus diseases, common cold, asthma, gastroesophageal reflux disease, bronchitis (e.g. acute bronchitis, chronic bronchitis, acute exacerbation of chronic bronchitis, or nonasthmatic eosinophilic bronchitis), bronchiectasis, post infectious cough, or others [12]. Thus, it is unexpectedly challenging for clinicians to define the etiology and pathogenesis of coughs in addition to selecting the optimal treatment in primary care settings. According to our knowledge, the present study incorporates the largest population sample yet to study the clinical epidemiology of coughs, which in turn can provide an overall figure indicating personal feelings, signs and clinical indications of coughs in primary care settings.

Subjects recruited in the present study had daytime or night symptoms of cough and diagnoses of respiratory disease, of whom about half were children. The accompanied symptoms included expectoration, fever, chest pain, usually indicative of the respiratory infection, wheezing, and breathlessness, which was usually accompanied with COPD and asthma. The two common diseases were COPD and asthma including cough variant asthma. Breathlessness and cough may be the clinical indicators of COPD or asthma exacerbations [13], while acute bronchitis should not be diagnosed until the common cold, asthma, and an acute exacerbation of chronic bronchitis have been ruled out [14]. However, it was often over-diagnosed, leading to the abuse of antibiotics. About 10% of patients were diagnosed as acute bronchitis in the Surveys, which was a lower percentage than that of COPD and asthma. Nasal disease was an important risk factor of cough, especially of chronic cough. It would be more valuable for the therapeutic strategies to further distinguish acute and chronic cough, rhinitis and other nasal disease if the proportion was large enough.

Hypersusceptibility has been suggested as one of major challenging factors in cough. We found that more than half of the patients with cough had a history of allergic rhinitis, asthma, allergic conjunctivitis or atopic dermatitis. About 15% to 23% patients had hyper-susceptibility, while 6% to 17% had a family history of hyper-susceptibility. More patients had a history of asthma in Survey 2 (64%) than in Survey 1 (39%). Allergic rhinitis is a common cause of postnasal drip and coughs, with a high prevalence in patients with asthma [15]. Patients with allergic rhinitis should be identified and clarified in the future study, in order to evaluate the significance and difference of allergic diseases in cough.

Procaterol was used as the treatment of patients, although the symptom of acute cough was sometimes automatically cured [16]. Responses to the used treatment were rapid and efficient, evidenced by the fact that 83% and 92% of patients in Surveys 1 and 2, respectively, had no daytime symptoms and 82% and 94% had no night symptoms after 14 days of treatment. Although our data showed that Procaterol could improve the symptom of cough in patients with respiratory diseases, we do not know the exact effects of the drug as our study was not a randomized controlled trial. Antibiotics were hardly effective for chronic cough and acute cough caused by the common cold, acute bronchitis, asthma, mild exacerbations of chronic bronchitis related to smoking, or environmental irritants [17,18]. However, the application of antibiotics could be beneficial for upper airway cough syndrome resulted from bacterial sinusitis, and infection in the lower respiratory tract (whooping cough), if given early. The prescription rate of antibiotics was about 4-6% in patients with cough, which was considered as acceptable according to the conditions of the patients studied. Inhaled steroids were used in about 2% of patients, while systemic corticosteroids in 0.6%.

However, a number of limitations in the present study should be improved in future studies. For example, such cross-sectional studies should be followed up longitudinally, results based on self-reports should be compared with clinical information, including physical examinations, imaging and biochemical analyses. More diagnoses should be included in the questionnaire form, including pneumonia, common cold, acute bronchitis, and chronic bronchitis, even though it may be difficult to confirm those diagnoses. More attention on etiology and pathogenesis of cough should be considered as well as further education on cough management should be delivered to physicians working in primary care settings.

## Conclusion

In conclusion, cough causes a high level of morbidity in the community, annually resulting in massive health economy consequences. Demography and symptomatology seem to be similar to that reported in specialist hospital centers, but successful and precise diagnosis and treatment of cough was not as satisfactory. Management of cough in primary care needs to be improved in China.
